# Towards the Development of a Conceptual Framework of the Determinants of Pre‐Eclampsia: A Hierarchical Systematic Review of Social Determinants

**DOI:** 10.1111/1471-0528.18082

**Published:** 2025-02-19

**Authors:** Mai‐Lei Woo Kinshella, Kelly Pickerill, Sarina Prasad, Olivia Campbell, Jalila Devji, Lívia Vieira Lopes, Rosa Balleny, Terteel Elawad, Rachel Craik, Marie‐Laure Volvert, Hiten D. Mistry, Hannah Blencowe, Véronique Filippi, Peter von Dadelszen, Laura A. Magee, Marianne Vidler, the PRECISE Network

**Affiliations:** ^1^ Department of Obstetrics and Gynaecology BC Children's and Women's Hospital and University of British Columbia Vancouver British Columbia Canada; ^2^ Department of Women and Children's Health School of Life Course and Population Sciences, Kings College London London UK; ^3^ Department of Population Health Sciences College of Life Sciences, University of Leicester Leicester UK; ^4^ Faculty of Epidemiology and Population Health London School of Hygiene & Tropical Medicine London UK

## Abstract

**Background:**

Existing reviews of pre‐eclampsia determinants have focused on clinical and genetic risk factors.

**Objective:**

To evaluate social determinants for pre‐eclampsia prevention.

**Search Strategy:**

Systematic searches were conducted from relevant electronic databases from inception of each database to 30th December 2024.

**Selection Criteria:**

Reviews and large cohort studies (≥ 1000 participants), published between 2013 and 2024, reporting quantitative associations between social determinant exposures and pre‐eclampsia outcomes.

**Data Collection and Analysis:**

Titles and abstracts, then relevant full‐texts were reviewed by two reviewers, independently. Strength of association was evaluated as ‘definite’ (odds ratios [OR] or relative risk [RR] ≥ 3.00 or < 0.33), ‘probable’ (OR or RR 1.50–2.99 or 0.33–0.67), ‘possible’ (OR or RR 1.10–1.49 or 0.68–0.89), or ‘unlikely’ (OR or RR 0.90–1.09). Quality of the evidence was high, moderate, low, or very‐low, using GRADE.

**Main Results:**

Twenty‐seven publications found 24 associations of pre‐eclampsia with socioeconomic status, social support/exclusion, healthcare access, and occupational and physical environmental factors. One association (polygamy) was definite (low‐quality evidence). Probable associations included: work stress, lack of antenatal care and heat exposure in early pregnancy (high‐quality evidence); prolonged occupational exposure to whole body vibrations or bending, distance to health facility, and UV‐B radiation exposure (protective factor), all based on moderate‐quality evidence; and neighbourhood deprivation, rotating work shifts, and Asian/Oceanian origins (protective factor), all based on low‐quality evidence. There were 13 possible associations, which did not include education.

**Conclusion:**

Our findings support recommendations to address climate change, strengthen occupational protection, and promote early antenatal attendance. Social determinants may be indicative of upstream factors (e.g., obesity) that increase likelihood of clinical risk factors for pre‐eclampsia incidence and severity.

## 1 Introduction

Pre‐eclampsia is a serious complication of pregnancy, characterised by new‐onset hypertension at or after 20 weeks' gestation with one or more of: proteinuria, maternal end‐organ involvement, or uteroplacental dysfunction [1]. Pre‐eclampsia is the second leading cause of maternal mortality worldwide, associated with over 46 000 maternal and 500 000 perinatal deaths annually [1]. This healthcare burden is disproportionately associated with resource‐limited settings and economically‐deprived communities, which highlights the need to consider the social environments where pregnant women develop the disease [2].

Social determinants of health are the non‐medical factors that influence health outcomes [3] and include social or environmental factors that contribute to, or detract from, the ability to live a healthy life [4]. The Royal College of Obstetricians and Gynaecologists (2022), recently highlighted how socioeconomic status (SES), employment status, educational status, migration, ethnicity, health service factors, and physical environmental factors can contribute to maternal morbidity and mortality [4].

Despite the growing body of literature on the social determinants of maternal health [5, 6, 7, 8, 9, 10], reviews of risk factors for pre‐eclampsia have largely focused only on clinical and genetic risk factors [11, 12, 13, 14, 15]. This evidence review aims to compile current literature on the social determinants of pre‐eclampsia, with any associations evaluated for their strength and the quality of the underlying evidence.

## 2 Methods

### 2.1 Search Strategy

Our review employed the methods of Hiatt et al., to develop a model of determinants using a systematic process [16], and is part of the larger aim of building a conceptual framework to describe a comprehensive multi‐factorial model of the determinants of pre‐eclampsia. A broad working model of known determinants were assembled by the ‘PREgnancy Care Integrating translational Science, Everywhere’ (PRECISE) Network [17], based on variables found to have significant associations with pre‐eclampsia from pooled results within umbrella reviews of systematic reviews [14, 15]; however, social determinants of health were inadequately covered in these reviews. Informed by four prominent social determinant of health frameworks: Healthy People 2030 [18], the World Health Organisation [19], Public Health Agency of Canada [20], and the Dahlgren‐Whitehead Rainbow model [21] (Table S2), we completed a search of social indicators, grouped into eight domains (SES, education, social support and exclusion, ethnicity and region of origin, occupation, healthcare access, and physical environment).

The search strategy was conducted by a team of researchers, graduate and undergraduate students and medical trainees (KP, OC, SP, MWK, JD, LVL, RD). Systematic searches were conducted on Medline, Embase, Health Technology Assessments, Database of Abstracts of Reviews of Effects, Cochrane Library, Google Scholar and reference lists. Searches were conducted for relevant articles in each domain, initially to 31st July 2023, with an update undertaken to 30th December 2024. Broad search terms are reported in Table 1. (File S1).

**TABLE 1:** Search terms.

Socioeconomic status, income, social gradient, poverty, deprivation	AND	Pre‐eclampsia
Education, illiteracy, literate		
Social capital, social support, community funds, women groups, social isolation, marital status, same‐sex marriage, refugees, immigrant, migrant, housing instability		
Ethnicity, region of origin, nativity		
Occupation, occupational exposure, work exposure, work stress		
Religion, Christian, Muslim, Islam, Hindu, Buddhism, agnostic, atheist		
Health services accessibility, prenatal care, rural health services, health facility density, skilled birth attendant, homebirth, private healthcare, distance to health, rural, money for transport		
Environmental pollution, air pollution, water pollution, noise, seasons, seasonality, temperature, humidity, rain, sunlight, climate change, physical environment		

### 2.2 Eligibility Criteria

We included recent studies published between 2013 and 2024 that reported quantitative associations between social determinant exposures and pre‐eclampsia. Our outcome of interest was pre‐eclampsia incidence, as defined according to the individual publications reviewed. Studies that only reported pregnancy hypertension or hypertension outside of pregnancy were excluded. Following methods of Hiatt et al. [16], studies were selected according to a hierarchy of evidence that prioritised umbrella reviews (systematic reviews of reviews), followed by systematic reviews with meta‐analyses and finally large observational cohort studies with a minimum sample size of 1000 pregnancies, as described by Bartsch et al. [11] to be more representative of the general population and to have sufficient statistical power to assess less prevalent, but potentially important, risk factors; we excluded smaller observational cohort studies, cross‐sectional surveys, and case reports or series, which may be less representative of the population of interest, Additionally, qualitative reviews and editorials were excluded. Studies conducted in any country were eligible for inclusion.

### 2.3 Study Selection and Data Extraction

Titles and abstracts of search results were screened by the search team (KP, OC, SP, MWK, JD, LVL, RD) to determine potential eligibility. All potentially eligible studies independently underwent full‐text review by two team members.

Data abstracted were general study characteristics, and strength of association between each social determinant and pre‐eclampsia, expressed as relative risk (RR), odds ratios (OR), or hazard ratio (HR), unadjusted and adjusted if reported, or calculated from raw data provided.

### 2.4 Certainty of the Evidence

All indicators were assessed by the quality of the evidence and the strength of association. Evidence quality was rated independently by two reviewers, using Grading of Recommendations, Assessment, Development, and Evaluation (GRADE) [22]. Following GRADE procedures, umbrella or systematic reviews were classed as high quality, while single observational studies were considered low quality that could be upgraded for large effect sizes or evidence of a dose response [23]. Evidence quality was downgraded for risk of bias, inconsistency (substantial heterogeneity *I* ^2^ > 50%), indirectness (results not reported for pregnant populations), imprecision (wide confidence intervals) and publication bias (funnel plot asymmetry, Egger's Test with *p* < 0.05). Directness and precision were supported by our eligibility criteria, as studies not conducted with pregnant populations and studies < 1000 were excluded. The strength of association was classified as definite (OR or RR ≥ 3.00 or < 0.33), probable (OR or RR 1.50–2.99 or 0.33–0.67), possible (OR or RR 1.10–1.49 or 0.68–0.89), or unlikely (OR or RR crosses one, or point estimate is in non‐significant range, 0.90–1.09), adapted from Hiatt et al. [16]. Study countries were categorised as high, upper‐middle, and low‐ or low‐middle income countries according to World Bank classifications. Findings were discussed with six patient partners from the REACH BC Registry and the Preeclampsia Foundation.

## 3 Results

**FIGURE 1:**
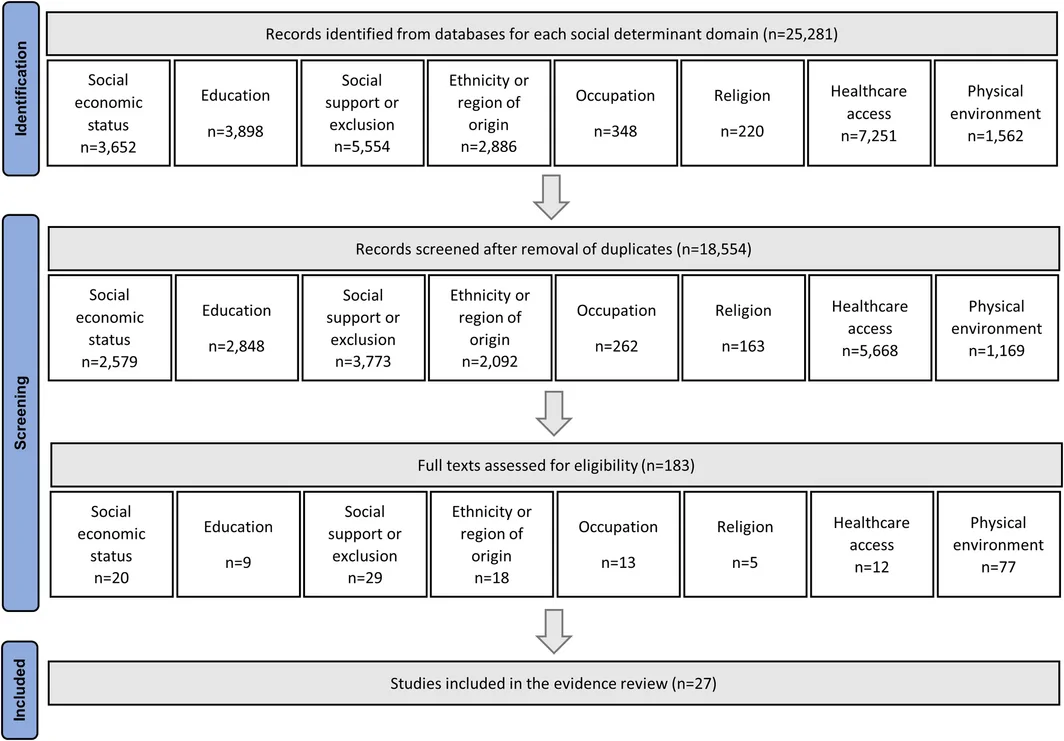
Search results.

Searches across the eight domains yielded 25 281 records. After removal of duplicates and screening of titles and abstracts, and review of 183 full‐texts (Figure 1), we included 27 articles. Included studies were primarily large individual cohort studies (*n* = 18) [24, 25, 26, 27, 28, 29, 30, 31, 32, 33, 34, 35, 36, 37, 38, 39, 40, 41] or systematic reviews with meta‐analyses (*n* = 8) [42, 43, 44, 45, 46, 47, 48, 49], although there was one umbrella review [14] Cohort studies had an average of 831 364 participants (range: 2492–5 448 255) and were primarily from Europe (*n* = 7) and North America (*n* = 7), with representation from South America (*n* = 1), Asia (*n* = 1), the Middle East (*n* = 1) and sub‐Saharan Africa (*n* = 1) (Table 2; Table S3; Figure S1). Meta‐analyses contributed 25 comparisons with an average of 1 878 638 participants in each (range: 3490–30 310 610) and an average of 5 studies in each comparison (range: 2–22) (Table 2). Of the 137 studies included in the 25 comparisons by meta‐analyses, most studies included in the systematic reviews were also from Europe (*n* = 35) and North America (*n* = 48), though there was more representation from Asia (*n* = 19), the Middle East (*n* = 12) and sub‐Saharan Africa (*n* = 19) (Table S3; Figure S1). South America (*n* = 2) and Australasia (*n* = 1) were minimally represented. Pre‐eclampsia was defined as high blood pressure (≥ 140/90 mmHg) after 20 weeks gestation with proteinuria, with proteinuria and other signs of organ damage, or with ICD 9/10 codes, all of which define pre‐eclampsia by high blood pressure and proteinuria (File S2).

**TABLE 2:** Social determinants examined for any association with risk of developing pre‐eclampsia (any, unless otherwise stated).

Category and indicators	Effect estimate (95% CI)	*N* studies	Country^a,b^	*N* participants	*I* ^2^	Direction of association	Strength of association	Certainty of evidence
Socioeconomic status								
Higher household income (vs lower)	OR 0.88 (0.86–0.89)	1	United States	5 448 255	NA	Protective	Possible	Low
Higher neighbourhood deprivation	RR 1.52 (1.35–1.71)	1	United Kingdom	159 125	NA	Risk factor	Probable	Low
Absence of standard health insurance	OR 1.98 (1.46–2.67)	1	Korea	461 580	NA	Risk factor	Probable	Low
Maternal education								
Low maternal education	OR 1.12 (0.59–1.65)	7^c^	Cameroon^a^, Mozambique^a^, Nigeria^a^ (2 studies), Sudan^a^, Tanzania^a^, Uganda^a^	4429	80%	None	Unlikely	Moderate
Social support and exclusion								
Polygamy	RR 3.13 (2.51–3.90)	1	Israel	9872	NA	Risk factor	Definite	Low
Immigrant status (vs native origin)	OR 0.75 (0.69–0.80)	22^c^	Australia, Canada, Chile, Greece, Israel, Norway (4 studies), Portugal (2 studies), Sweden (2 studies), Turkey^*†*^ (6 studies), United States (3 studies)	30 310 610	93%	Protective	Possible	Moderate
Refugee status	OR 1.62 (1.55–1.69)	1	Ecuador^b^	1 165 801	NA	Risk factor	Possible	Low
Specifically for preterm pre‐eclampsia (< 37 weeks)	OR 1.18 (1.02–1.36)	1	Norway	1 287 270	NA	Risk factor	Possible	Low
Specifically for very preterm pre‐eclampsia (< 34 weeks)	OR 1.41 (1.15–1.72)	1	Norway	1 287 270	NA	Risk factor	Possible	Low
Unmarried marital status (vs married)	OR 1.21 (1.11–1.32)	1	United States	205 888	NA	Risk factor	Possible	Low
Specifically for early‐onset (< 34 weeks) pre‐eclampsia	HR 1.14 (1.10–1.19)	1	United States	456 668	NA	Risk factor	Possible	Low
Specifically for late‐onset (≥ 34 weeks) pre‐eclampsia	HR 1.14 (1.10–1.19)	1	United States	456 668	NA	Risk factor	Possible	Low
Mental stress	OR 1.49 (1.27–1.74)	12^d^	Canada (2 studies), Finland, Germany, Iran^b^, Netherlands, Nigeria^a^, Pakistan^a^, Peru^b^, United States (3 studies)	665 893	68%	Risk factor	Possible	Low
Unstable housing	OR 1.0 (0.7–1.3)	1	United States	2 898 035	NA	None	Unlikely	Very low
Ethnicity & region of origin								
Asia and Oceania (vs. Swedish European)	RR 0.60 (0.47–0.75)	1	Sweden	46 618	NA	Protective	Probable	Low
Africa (vs Swedish European)	RR 1.05 (0.69–1.58)	1	Sweden	46 618	NA	None	Unlikely	Very low
Americas (vs Swedish European)	RR 1.00 (0.65–1.53)	1	Sweden	46 618	NA	None	Unlikely	Very low
European Union countries (vs Swedish European)	RR 0.97 (0.73–1.29)	1	Sweden	46 618	NA	None	Unlikely	Very low
Other European countries (vs Swedish European)	RR 0.89 (0.69–1.14)	1	Sweden	46 618	NA	None	Unlikely	Very low
Non‐Hispanic Black (vs. Non‐Hispanic White)	OR 1.17 (0.87–1.56)	1	United States	6096	NA	None	Unlikely	Low
Hispanic (vs. Non‐Hispanic White)	OR 1.16 (0.84–1.59)	1	United States	6096	NA	None	Unlikely	Very low
Occupation								
Work stress	OR 1.50 (1.15–1.97)	4^d^	Canada (2 studies), Netherlands, United States	8742	0%	Risk factor	Probable	High
Whole body vibrations (highest [≥ 0.5 m/s^2^] vs. no exposure 0 m/s^2^)	OR 1.76 (1.41–2.20)	1	Sweden	646 490	NA	Risk factor	Probable	Moderate
Prolonged bending (≥ 1 h/day)	OR 1.51 (1.09–2.08)	2^c^	Canada, Norway	9970	12%	Risk factor	Probable	Moderate
Rotating shifts	OR 1.75 (1.01–3.01)	2^c^	Norway, Taiwan	29 588	75%	Risk factor	Probable	Low
Heavy lifting (≥ 11 kg/time)	OR 1.35 (1.07–1.71)	5^c^	Canada, Netherlands, Norway, United States (2 studies)	20 716	0%	Risk factor	Possible	Moderate
Passive job strain: within a low‐demand job, low (vs high) decision‐making authority	RR 1.10 (1.06–1.14)	1	Sweden	1 102 230	NA	Risk factor	Possible	Low
Active job strain: within high decision‐making authority, high (vs low) demand job	RR 0.90 (0.87–0.94)	1	Sweden	1 102 230	NA	None	Unlikely	Low
High job strain: low decision‐making authority and high demand job (vs high decision authority and low demand)	RR 1.02 (0.98–1.06)	1	Sweden	1 102 230	NA	None	Unlikely	Low
Long working hours (> 40 h/week)	OR 1.27 (0.74–2.19)	5^c^	Canada (2 studies), Netherlands, Taiwan, United States	34 650	84%	None	Unlikely	Low
Overnight shifts	OR 1.05 (0.63–1.75)	3^c^	Canada, Netherlands, Taiwan	33 247	0%	None	Unlikely	Moderate
Occupational noise exposure	RR 1.07 (1.04–1.10)	4^c^	Canada, Norway, Sweden, United States	1 101 240	41%	None	Unlikely	Moderate
Prolonged standing (> 4 h/day)	OR 0.95 (0.58–1.55)	6^c^	Canada (2 studies), Netherlands, Norway, United States (2 studies)	26 831	78%	None	Unlikely	Low
Prolonged or walking (> 4 h/day)	OR 0.70 (0.46–1.08)	3^c^	Canada (2 studies), Netherlands	9777	41%	None	Unlikely	Moderate
Heavy physical workload	OR 1.30 (0.69–2.43)	2^c^	Italy, United States	6085	3%	None	Unlikely	Moderate
Healthcare access								
No ANC visits (vs at least one visit)	OR 2.71 (1.45–3.96)	6^c^	Ethiopia^a^, Nigeria^a^, Sudan^a^ (3 studies), Uganda^a^	3490	93%	Risk factor	Probable	High
24 h MFM specialist coverage (vs no 24 h specialist coverage)	OR 1.49 (1.01–2.19)	1	United States	205 888	NA	Risk factor	Possible	Low
Tertiary‐level teaching hospital (vs community hospital)	OR 0.78 (0.33–1.84)	1	United States	205 888	NA	None	Unlikely	Low
Distance to health facility (> 30 miles/50 km)	OR 1.72 (1.23–2.45)	1	United States	2492	NA	Risk factor	Probable	Moderate
Rural residence	OR 0.91 (0.62–1.33)	1	United States	2492	NA	Risk factor	Unlikely	Very low
Specifically for eclampsia	OR 2.70 (1.80–4.07)	1	Canada	256 220	NA	Risk factor	Probable	Low
Physical environment								
*Ambient temperature*								
Heat exposure in early pregnancy (1–20 weeks gestation)	OR 1.54 (1.10–2.15)	4^c^	Canada, China^b^, Israel, South Africa^b^	4 006 445	99%	Risk factor	Probable	High
Heat exposure in late pregnancy (1–20 weeks gestation)	OR 1.05 (0.67–1.64)	4^c^	Canada, China^b^, Israel, South Africa^b^	1 964 476	96%	None	Unlikely	Low
Cold exposure in early pregnancy (> 20 weeks gestation)	OR 0.90 (0.84–0.97)	4^c^	Canada, China^b^, Israel, South Africa^b^	4 006 445	66%	None	Unlikely	Moderate
Cold exposure in late pregnancy (> 20 weeks gestation)	OR 1.13 (0.84–1.53)	4^c^	Canada, China^b^, Israel, South Africa^b^	1 964 476	87%	None	Unlikely	Low
High (vs. lowest) exposure to UV‐B radiation	OR 0.57 (0.44–0.72)	1	Scotland	522 896	NA	Protective	Probable	Moderate
High (vs. lowest) exposure to sunlight	OR 0.77 (0.62–0.95)	1	United States	205 888	NA	Protective	Possible	Low
Precipitation (rainy vs. dry season)	OR 1.36 (1.11–1.65)	1	Rwanda^a^	19 746	NA	Risk factor	Possible	Very low
Noise pollution (65 vs. 50 dB)	OR 1.09 (0.99–1.20)	1	Canada	269 263	NA	None	Unlikely	Low
Specifically for preterm pre‐eclampsia (< 34 weeks)	OR 1.71 (1.20–2.43)	1	Canada	269 263	NA	Risk factor	Probable	Moderate
*Outdoor* (*ambient*) *air pollution*								
Air pollution (NO_2_)	OR 1.10 (1.03–1.17)	4^d^	Australia, Netherlands, Spain, United States	121 126	0%	Risk factor	Possible	High
Nitrogen oxides (NO_x_)	OR 1.03 (0.91–1.17)	3^d^	Spain, Sweden, United States	50 110	0%	None	Unlikely	Moderate
Ozone (O_3_)	OR 1.10 (0.99–1.22)	3^d^	Sweden, United States (2 studies)	115 891	24%	None	Unlikely	Moderate
Carbon monoxide (CO)	OR 1.03 (1.00–1.06)	3^d^	Canada, United States (2 studies)	169 303	0%	None	Unlikely	High
PM_10_	OR 1.11 (1.03–1.21)	12^d^	China (4 studies), Korea, Netherlands, Spain, Sweden, United States (4 studies)	1 729 656	82%	Risk factor	Possible	Moderate
PM_2.5_	OR 1.12 (1.00–1.25)	10^c^	China (4 studies), Spain, Sweden, United States (4 studies)	534 430	69%	None	Unlikely	Low
Indoor (household) air pollution	OR 1.10 (0.54–2.22)	3^c^	Ghana^a^, India^a^ (2 studies)	38 315	79%	None	Unlikely	Very low

Abbreviations: ANC, antenatal care; dB, decibels; h, hours; HELLP, haemolysis, elevated liver enzymes, low platelets syndrome; MFM, maternal fetal medicine; NO_2_, nitrogen dioxide; PM, particulate matter; UV‐B, ultraviolet B.

^a^ A low or lower‐middle income country.

^b^ An upper‐middle income country according to the World Bank; All others are high‐income countries.

^c^ *N* studies in a systematic review(s).

^d^ *N* studies in an umbrella review.

Overall, 66 indicators were evaluated across the eight domains, with the following associations observed with pre‐eclampsia incidence: 24 indicators with definitive, probably, or possible associations (Table 2); 27 indicators that were unlikely to be associated; and 15 indicators for which there was insufficient evidence to make an assessment, according to our methodology (Files S1 and S2). GRADE assessments for all indicators are reported in File S3.

### 3.1 Socioeconomic Status

SES was evaluated by three large cohort studies from the United States, the UK and Sweden (6 068 960 participants total), all assessed as providing low‐quality evidence (Table 2). The association with pre‐eclampsia was: ‘probable’ for lower SES defined by the absence of standard health insurance [25] or higher levels of neighbourhood deprivation [26], and higher household income was ‘possible’ protective factor [24].

### 3.2 Maternal Education

A meta‐analysis of seven studies (4429 participants) from sub‐Saharan Africa found that low maternal education was not associated with higher odds of pre‐eclampsia (OR 1.12, 95% CI: 0.59–1.65, *I* ^2^ 80%) [42], but the finding was downgraded to moderate‐quality evidence due to high between‐study heterogeneity in outcomes (i.e., *I* ^2^ = 80%).

### 3.3 Social Support and Exclusion

The association between polygamy and the risk of pre‐eclampsia was rated as ‘definite’ [29], but the quality was low, due to evidence availability from only one country in the Middle East.

Recent immigration was a ‘possible’ protective factor for pre‐eclampsia, based on a meta‐analysis of 24 studies (30 310 610 participants), but the evidence quality was moderate based on very high heterogeneity (*I* ^2^ 93%) [43]. Based on a large cohort study from Ecuador (1 154 891 participants), refugee status was a ‘probable’ risk factor, based on low‐quality evidence [30]. Another cohort study from Norway reported that refugee status was specifically a ‘possible’ risk factor for preterm or very preterm pre‐eclampsia [31].

Data informing the association between marital status and pre‐eclampsia come from two large cohort studies (662 556 participants) undertaken in the United States. Being unmarried (vs. married) is a ‘possible’ risk factor for pre‐eclampsia, based on low‐quality evidence [27], as well as for early‐onset (< 34 weeks) or late‐onset (≥ 34 weeks) pre‐eclampsia, again based on low‐quality evidence [28].

Mental stress is another ‘possible’ risk factor for pre‐eclampsia, based on an umbrella review that included 12 studies (665 893 participants), assessed with low‐quality evidence due to high heterogeneity (*I* ^2^ 68%) and potential publication bias (Egger's Test 0.02) [14].

Unstable housing was not associated with pre‐eclampsia in a large cohort study from the United States (665 893 participants) providing very low‐quality evidence [32].

### 3.4 Ethnicity and Region of Origin

Most ethnicities and region of origin were not significantly associated with pre‐eclampsia risk.

Only Asia and Oceania region of origin was protective against pre‐eclampsia incidence in a cohort study conducted in Sweden (RR 0.60, 95% CI: 0.47–0.75, 46 618 participants), while other global regions did not have significantly different rates from Swedish European, after adjusting for confounders [33].

An American cohort study did not find significant differences between Non‐Hispanic Black (OR 1.17, 95% CI: 0.87–1.56, 6096 participants) or Hispanic (OR 1.16, 95% CI: 0.84–1.59, 6096 participants) compared with Non‐Hispanic White pregnant people after adjusting for confounders [34]. A lack of published meta‐analyses on ethnicity and region of origin contributed to low‐/very low‐quality of the evidence.

### 3.5 Occupation

There were numerous occupational risk factors for pre‐eclampsia. ‘Probable’ associations included work stress (8742 participants, high‐quality) from an umbrella review [14], prolonged bending for at least 1 h/day (9970 participants, moderate‐quality) [45], and rotating shifts (29 588 participants, low‐quality) [44] from two systematic reviews, and occupational exposure to whole body vibrations (moderate‐quality) reported in a Swedish cohort study (646 490 participants) [35]. Heavy lifting of 11 kg or more at any one time (20 716 participants, moderate‐quality) was evaluated as a ‘possible’ risk factor in a systematic review [45], and passive job strain, meaning within the context of low decision‐making authority, particularly with low demand (e.g., no tight deadlines, high targets, or conflicting pressures) was evaluated as a ‘possible’ risk factor in a Swedish cohort study (1 102 230 participants, low‐quality) [36]. Evaluated as ‘unlikely’ risk factors for pre‐eclampsia were: high‐ (vs. low‐) demand job within high decision‐making authority or high‐demand/low decision‐making (vs low‐demand/high decision‐making) job [36], as well as long working hours (> 40 h/week), overnight shifts, occupational noise exposure, prolonged standing or walking (> 4 h/day), and heavy physical workload (all from meta‐analyses [44, 45, 46], low/moderate quality).

### 3.6 Healthcare Access

Having no antenatal care (ANC) visits (vs at least one visit) was a ‘probable’ risk factor for pre‐eclampsia in a meta‐analysis of six African studies (3490 participants, *I* ^2^ 93%) (high‐quality evidence, downgraded for high heterogeneity, but upgraded for large effect size) [42]. Having access to 24‐h maternal‐fetal medicine specialist coverage was a ‘possible’ risk factor, although rates did not significantly differ between hospital levels (tertiary‐level teaching hospital vs. community hospital) in one study conducted in the United States [27]. A cohort study from the United States (2492 participants) found that over 30 miles (50 km) distance to health facility was probable risk factor, based on moderate‐quality evidence, though rural residence was an unlikely risk factor [37]. While rural residence may not be associated with higher odds of pre‐eclampsia, another cohort study from the United States found a probable association with eclampsia, though based on low‐quality evidence [38].

### 3.7 Physical Environment

Heat exposure in early pregnancy (week 1–20 gestation) was ‘probable’ risk factor for pre‐eclampsia in a meta‐analysis of four studies from Canada, China, Israel and South Africa (4 006 445 participants, *I* ^2^ 99%) (high‐quality evidence, heterogeneity upgraded for dose response: every 1°C temperature increase in early pregnancy OR 1.07, 95% CI: 1.02–1.12) [47]. Heat exposure in late pregnancy and cold exposure in early or late pregnancy were ‘unlikely’ risk factors based on moderate‐low quality evidence [47].

Sunlight (solar radiation) was found to be a protective factor for pre‐eclampsia, with associations that were ‘probable’ when exposure was UV‐B radiation (moderate‐quality, American cohort study with 205 888 participants), and ‘possible’ when there was direct exposure to sunlight (low‐quality, cohort study from Scotland with 522 896 participants) [27, 39].

The rainy season was a ‘possible’ risk factor for pre‐eclampsia (very low‐quality), based in a cohort study from Rwanda (19 746 participants) [40].

Environmental noise pollution (neighbourhood exposure to 65.0 dB vs. 50 dB) was a risk factor only for very preterm pre‐eclampsia (moderate‐quality), but not pre‐eclampsia overall (low‐quality) in a Canadian cohort study (269 263 participants) [41].

Outdoor (ambient) air pollution consisting of nitrogen dioxide (NO_2_) (high‐quality, umbrella review) [14] and particulate matter PM_10_ (moderate‐quality, systematic review) during pregnancy [48] were ‘possible’ risk factors for pre‐eclampsia. However, evaluated as ‘unlikely’ was a relationship between pre‐eclampsia and other components of indoor (household) and outdoor air pollution, including: nitrogen oxides (NO_x_), ozone (O_3_), carbon monoxide (CO), and particulate matter PM_2.5_; all evaluated from systematic reviews with meta‐analyses [14, 48, 49].

### 3.8 Results by Country Income Status

Table 3 reports a summary of associations by evidence quality, strength, and country income status. 71% (17 of 24) of indicators with significant associations in this review drew on studies conducted in HICs. Of the six other indicators, one risk factor (refugee status) was studied in an upper‐middle income country, two risk factors (lack of ANC visits and rainy season) were studied in low‐ or low‐middle income countries, and the risk factors of mental stress and heat exposure in early pregnancy, and immigrant status as a protective factor, were based on meta‐analyses that included studies with multiple country income classifications. Of the 27 indicators with unlikely associations with pre‐eclampsia incidence, 81% [22] were from studies conducted in HICs. Maternal education and indoor (household) air pollution were unlikely risk factors based on studies conducted in low‐ or low‐middle income countries; heat exposure in late pregnancy, and cold exposure in early or late pregnancy, were unlikely risk factors based on meta‐analyses that included studies from multiple country income classifications.

**TABLE 3:** Summary of social determinants of pre‐eclampsia by evidence quality and strength.

		Quality of the evidence			
		High	Moderate	Low	Very Low
Strength of the association	Definite			Polygamy^c^	
	Probable	Work stress^c^ Lack of antenatal care visits^a^ Heat exposure in early pregnancy^d^	Occupational exposures to whole body vibrations^c^ Occupational exposure to prolonged bending^c^ Distance to health facility^c^ UV‐B exposure ^c^	Neighbourhood deprivation^c^ Asia and Oceania region of origin ^c^ Occupational exposure to rotating shifts^c^	
	Possible	Air pollution (NO_2_)^c^	Immigrant status ^d^ Occupational exposure to heavy lifting^c^ PM_10_ ^c^	Higher household income ^c^ Absence of standard health insurance^c^ Refugee status^b^ Unmarried marital status^c^ Mental stress^d^ Passive job strain^c^ 24 h MFM specialist coverage^c^ Sunlight exposure ^c^	Rainy season^a^
	Unlikely	Carbon monoxide (CO)^c^	Maternal education level^a^ Occupational exposure to overnight shifts^c^ Occupational noise exposure^c^ Occupational exposure to prolonged walking^c^ Heavy physical workload^c^ Cold exposure in early pregnancy^d^ Nitrogen oxides (NO_x_)^c^ Ozone (O_3_)^c^	Non‐Hispanic Black^c^ Active job strain^c^ High job strain^c^ Occupational exposure to long working hours^c^ Occupational exposure to prolonged standing^c^ Tertiary‐level teaching hospital^c^ Heat exposure in late pregnancy^d^ Cold exposure in late pregnancy^d^ Noise pollution^c^ PM_2.5_ ^c^	Unstable housing^c^ Africa region of origin^c^ Americas region of origin^c^ European Union region of origin^c^ Other European region of origin Hispanic^c^ Rural residence^c^ Indoor (household) air pollution^a^

*Note:* Protective factor.

Abbreviations: MFM, maternal‐fetal medicine; PM, particulate matter.

^a^ Evidence from low or low‐middle income countries.

^b^ Evidence from upper‐middle income countries.

^c^ Evidence from high income countries.

^d^ Evidence from mixed country income categories.

## 4 Discussion

### 4.1 Summary of Findings

Our hierarchical systematic review found that access to care (lack of ANC and distance to health facility), occupational conditions (work stress, full body vibrations, and prolonged bending), and environmental conditions (exposure to elevated ambient temperatures in early pregnancy) have probable associations with pre‐eclampsia, based on moderate‐high quality evidence, largely from HICs. Exposure to UV‐B radiation was the only protective factor found with probable association based on moderate quality evidence. Many associations were based on low quality evidence as assessed by GRADE, including SES (probable risk factors: neighbourhood deprivation and absence of standard medical insurance; possible protective factor: higher household income), social support (polygamy, unmarried marital status, mental stress, and refugee status risk factors) and region of origin (Asian and Oceanian origins as a protective factor), with the exception of immigrant status which was a possible protective factor based on moderate‐quality evidence. Evidence did not support associations with maternal educational levels and we identified a lack of relevant evidence for religion, based on our methodology.

The certainty of evidence of included studies is challenged by many associations based largely on individual cohort studies, including for SES, polygamy, refugee status, unmarried marital status and unstable housing, ethnicity and region of origin, rural residence and distance to maternity health facility, hospital level, and exposure to sunlight, precipitation and noise. Evidence from individual cohort studies limits the generalisability of findings as the health impacts of social and socioeconomic context may differ by region. Moreover, of the included cohort studies, only one was from a low‐ and middle‐income country, where the social determinants of health may both more variable and play a greater contributory part to the origins of pre‐eclampsia. Associations with low maternal education, immigrant status, mental stress, work stress and occupational hazards, ambient temperature and air pollution were supported with evidence from reviews, though still largely from high‐income countries, except for low maternal education and household air pollution. Certain determinants may have an adverse effect everywhere, such as the lack of access to adequate maternity care, poor working conditions and temperatures, while some determinants may be more dependent on context. For example, the effect of SES may vary between overall resources available in a high‐income country compared to a low‐income country, and experiences of polygamy may be culture‐specific.

### 4.2 Comparison With Current Literature

We found that SES was associated with pre‐eclampsia, but maternal education was not. Non‐significant associations between pre‐eclampsia and maternal education were reported both in sub‐Saharan Africa [42] and Sweden [33], suggesting a similar relationship in high‐ and low‐ and middle‐income countries. While studies have previously used maternal education as an indicator of SES, our findings suggest that this may be problematic [50, 51]. In fact, it has been demonstrated that economic indicators (e.g., wealth and family income) are most sensitive to health outcomes among women, particularly among those of reproductive age [52]. This may stem from societal gender inequities leading to women (compared with men) receiving lower income returns from a similar level of education, as well as different occupational opportunities [53]. Associations may also be driven by underlying medical risk factors, such as higher rates of overweight/obesity and early pregnancy blood pressure reported among pregnant people with lower educational backgrounds [51].

Similar to our findings, a previous systematic review about hypertensive disorders of pregnancy (HDPs) found that job strain and full body vibrations were risk factors for pre‐eclampsia, although many factors were understudied and there was heterogeneity between studies in definitions and findings [54]. Of note, occupational risk factors in resource‐limited settings are understudied in particular. Nevertheless, the aforementioned review included one study from Africa—a small case–control study from Nigeria—that found double the odds of pre‐eclampsia among women with a stressful work environment during pregnancy (aOR 2.10; 95% CI 1.20–3.71) [55].

Although Black and African‐American women are often reported to be disproportionately affected by pre‐eclampsia [56, 57, 58], we did not find a significant association between pre‐eclampsia and either African region of origin or non‐Hispanic Black ethnicity, perhaps due to the protective effect of immigration. Immigrant populations tend to have lower rates of pre‐eclampsia and other HDPs and the risk of pre‐eclampsia increases by length of residence for migrant women [34, 43, 58, 59, 60]. Second, ethnicity and region of origin differences may be driven by existing medical conditions [61]. While non‐Hispanic Black pregnant people initially appeared to have higher rates of pre‐eclampsia in comparison to non‐Hispanic White, the association was not significant after adding chronic hypertension, chronic diabetes, gestational diabetes, parity, smoking, and BMI to the statistical model [34]. These findings are consistent with a French study in which pre‐pregnancy obesity mediated the heightened risk of severe pre‐eclampsia among sub‐Saharan African immigrants [62]. Disparities between groups may be related to how experiences of systemic racism intersect with SES, obesity, and access to care [26, 56, 57, 63, 64, 65, 66, 67].

While this review sought primarily to compile social risk factors for pre‐eclampsia incidence, social inequities may also have a powerful impact on pre‐eclampsia severity. Higher rates of preterm pre‐eclampsia have been documented with refugee status [31], and higher rates of eclampsia and HELLP syndrome found with rural residency/greater distance to health facility [38, 68]. Poverty and deprivation have been associated with increased rates of eclampsia and higher blood pressure (systolic BP ≥ 160 mmHg) [69, 70] and significantly higher odds of blood transfusion ≥ 4 units and admission to intensive care among non‐Hispanic Black (vs. non‐Hispanic White) women diagnosed with HDP [71]. These differences may be related to access to care, which, as indicated by ANC visits, was one of the strongest social determinants we found. Across sub‐Saharan Africa, lower healthcare worker densities was associated with lower likelihood of ANC, as well as reduced urine and blood pressure checks for pre‐eclampsia [72]. Within a high‐income country setting, differential prenatal care has been documented between African immigrant and native French populations, including blood pressure measurement and proteinuria testing procedures which may have delayed timely diagnosis of hypertension or pre‐eclampsia among African immigrant groups [73]. Lack of access to standard health insurance has also been linked with more severe symptoms of pre‐eclampsia [70, 74].

Physical environmental risk factors for pre‐eclampsia around seasons, elevated temperatures and intense humidity, aligns with a growing body of literature on the potentially negative effects of climate change on pre‐eclampsia incidence [75]. While the heterogeneity of global weather patterns complicates meta‐analyses on seasonality, two reviews found an increased risk of pre‐eclampsia associated with maternal exposure to heatwaves and high average temperature, potentially associated with reduced placental weight and volume [76, 77]. A review and a large time‐to‐event study in South Africa found that high temperatures, especially in early pregnancy (i.e., 2–5 weeks gestation), were associated with an increased risk of pre‐eclampsia [77, 78]. The protective effect of sunlight exposure may be associated with synthesis of vitamin D, which is involved in the absorption of calcium, both associated with pre‐eclampsia prevention [79, 80, 81].

### 4.3 Strengths and Limitations

To our knowledge, this is the first peer‐reviewed evidence review on the social determinants of pre‐eclampsia. A particular strength of our review is the systematic process of developing a model of determinants, study selection, and the use of GRADE and strength of association assessment to ascertain certainty of evidence. While our hierarchical approach to literature review systematically identified higher‐quality evidence (from systematic reviews and large observational cohort studies), we accept that smaller observational studies were excluded, and this may have meant that some risk factors were not included in the final list of determinants of pre‐eclampsia; as such, a broader range of determinants may have been identified a more traditional systematic review, that would have included lower‐level evidence. Second, because large cohort studies were the primary type of report on which this evidence review was based, our review was limited by the availability and quality of evidence. As discussed, many reports originated from high‐income countries. Some indicators, such social support and racism, may be hard to measure quantitatively, as seen by many reports having only qualitative analyses. Substantial heterogeneity in how indicators were conceptualised, such as ethnicity, SES, and seasonality, made meta‐analyses difficult. A number of relevant findings were reported only as conference abstracts without further publication as manuscripts, such as maternal deprivation [69] and insurance status [70], suggesting potential publication biases within this field of study. Additionally, while systematic reviews along with other study designs included in our review were assessed using GRADE to ascertain quality of the evidence, meta‐analyses typically pool raw effect estimates from individual studies without accounting for potential confounders, and this may bias outcomes. Lastly, the current review did not examine indirect associations between social determinants or between social and clinical determinants, which will be investigated in a follow‐up review by the PRECISE Network.

## 5 Conclusion

Our hierarchical review of social determinants of pre‐eclampsia supports recommendations to address climate change and strengthen occupational protection globally, as well as encourage early ANC attendance. All such determinants are potentially modifiable at the individual‐level, with adequate knowledge and supportive labour policy for environmental exposures, and community awareness for early ANC attendance; clinicians should consider the modifiability of risk factors for pre‐eclampsia, including inequities in SES and social support that may underly medical risk factors. Additionally, social determinants may be indicative of upstream factors (such as obesity) that increase the likelihood of clinical risk factors for pre‐eclampsia, as well as its incidence and severity. While social determinants are critical to our understanding of pre‐eclampsia, our review has highlighted heterogeneity in definitions, evidence gaps, and the low quality of existing evidence, particularly given that many results were derived from single large cohort studies, which presents serious limitations in terms of geographical contexts. Further high‐quality research and meta‐analyses on social determinants of pre‐eclampsia are critically needed, especially studies in resource‐limited settings.

## Author Contributions

M.V., L.A.M., P.v.D. and V.F. conceptualised the review. M.‐L.W.K. and K.P. conducted the review with S.P., O.C., J.D., L.V.L. and R.B. M.‐L.W.K. and K.P. wrote the first draft of the manuscript. M.V., L.A.M., P.v.D., V.F., H.B., H.D.M., M.‐L.V., R.C. and T.E. reviewed all versions of the paper and contributed to the interpretation and the structure of the paper. All authors read and approved the final manuscript.

## Ethics Statement

The review only utilised data from previous published studies.

## Conflicts of Interest

The authors declare no conflicts of interest.

## Supporting information


**Figure S1.** Map of included study countries.
**Table S1.** The PRECISE Network.
**Table S2.** Social determinants of health frameworks.
**Table S3.** Number of studies by country.

**File S1.** Search terms and results.

**File S2.** Summary of findings.

**File S3.** GRADE assessments.

## Data Availability

The data that supports the findings of this study are available in the Supporting Information of this article.
