# Supplementary material for: Towards the Development of a Conceptual Framework of the Determinants of Pre-Eclampsia: A Hierarchical Systematic Review of Social Determinants
Source: BJOG. Author manuscript; Available in PMC 2025 Oct 3. (PMC7618191; doi:10.1111/1471-0528.18082)

**Supplementary Appendix**

|  | Title | Page number |
| --- | --- | --- |
| **Tables** |  |  |
| Table S1 | The PRECISE Network | 2 |
| Table S2 | Social determinants of health frameworks | 4 |
| Table S3 | Number of studies by country | 5 |
| **Figures** |  |  |
| Figure S1 | Map of included study countries | 6 |

**Table S1**: The PRECISE Network

| **In-country teams** | **Members** |
| --- | --- |
| THE GAMBIA: Medical Research Council Unit The Gambia at the London School of Hygiene and Tropical Medicine, Fajara | Umberto D’Alessandro, Anna Roca, Hawanatu Jah, Andrew Prentice, Melisa Martinez-Alvarez, Brahima Diallo, Abdul Sesay, Sambou Suso, Baboucarr Njie, Fatima Touray, Yahaya Idris, Fatoumata Kongira, Modou F.S. Ndure, Lawrence Gibba, Abdoulie Bah and Yorro Bah. |
| KENYA: Aga Khan University, Nairobi | Marleen Temmerman, Angela Koech, Patricia Okiro, Geoffrey Omuse, Grace Mwashigadi, Mary Goretti Amondi, Consolata Juma, Joseph Mutunga, Moses Mukhanya, Robin Okello, Onesmus Wanje, Isaac Mwaniki, Marvin Ochieng, Emily Mwadime |
| MOZAMBIQUE : Centro de Investigação em Saúde de Manhiça, Manhiça | Esperança Sevene, Corssino Tchavana, Salesio Macuacua, Anifa Vala, Helena Boene, Lazaro Quimice, Sonia Maculuve, Inacio Mandomando |
| **Central co-ordinating team** |  |
| Department of Women and Children’s Health, School of Life Course Sciences, Faculty of Life Sciences and Medicine, King’s College London | Peter von Dadelszen, Laura A. Magee, Rachel Craik, Marie-Laure Volvert, Hiten Mistry, Thomas Mendy |
| Donna Russell Consulting | Donna Russell |
| **Co-Investigator team** |  |
| Midlands State University, Zimbabwe | Prestige Tatenda Makanga, Liberty Makacha and Reason Mlambo |
| Kings College London | Lucilla Poston, Rachel Tribe, Sophie Moore, Tatiana Salisbury |
| University of Oxford | Aris Papageorghiou, Alison Noble, Rachel Craik |
| London School of Hygiene and Tropical Medicine | Hannah Blencowe, Veronique Filippi, Joy Lawn, Matt Silver, Joseph Akuze and Ursula Gazeley |
| St George’s, University of London | Judith Cartwright, Guy Whitley, Sanjeev Krishna |
| University of British Columbia | Marianne Vidler, Jing (Larry) Li, Jeff Bone, Mai-Lei (Maggie) Woo Kinshella, Domena Tu, Ash Sandhu, Kelly Pickerill |
| Eduardo Mondlane University, Maputo | Carla Carillho |
| Imperial College London | Benjamin Barratt |

**Table S2:** Social determinants of health frameworks

|  | **Healthy People 2030** | **World Health Organization** | **Public Health Agency of Canada** | **Dahlgren-Whitehead** |
| --- | --- | --- | --- | --- |
| **Socioeconomic status** | Economic stability | Social gradient | Income and social status | - |
| **Education** | Education | - | Education and literacy | Education |
| **Employment and work** | - | Unemployment | Employment/ working conditions | Unemployment |
|  |  | Work |  | Work environment |
| **Early life*** | - | Early life | Healthy child development |  |
| **Social support or exclusion** | Social and community context | Social support | Social support networks | Social and community networks |
|  |  | Social exclusion | Social environments |  |
| **Culture** | - | - | Culture | - |
| **Mental health*** | - | Stress | - | - |
| **Healthcare** | Health | Addiction | Personal health practices and coping skills | Individual lifestyle factors (age, sex, constitutional factors) |
|  | Health care |  | Health services | Health care services |
| **Gender** | - | - | Gender | - |
| **Genetics*** | - | - | Biology and genetic endowment | - |
| **Nutrition*** | - | Food |  | Agriculture & food production |
| **Transport** | - | Transport | - | - |
| **Physical environment** | Neighbourhood and built environment |  | Physical environment | WASH (water, sanitation, and hygiene |

** Part of the larger PRECISE Network conceptual framework, including sections specifically on clinical risk factors, biomarkers and nutrition.*

**Table S3:** Number of studies by country

| **Country** | **Meta-analysis*** | **Cohort studies** | **Total** |
| --- | --- | --- | --- |
| Australia | 2 |  | 2 |
| Cameroon | 1 |  | 1 |
| Canada | 20 | 2 | 22 |
| Chile | 1 |  | 1 |
| China | 15 |  | 15 |
| Ecuador |  | 1 | 1 |
| Ethiopia | 1 |  | 1 |
| Finland | 1 |  | 1 |
| Germany | 1 |  | 1 |
| Ghana | 1 |  | 1 |
| Greece | 1 |  | 1 |
| India | 2 |  | 2 |
| Iran | 1 |  | 1 |
| Israel | 5 | 1 | 6 |
| Italy | 1 |  | 1 |
| Korea | 1 | 1 | 2 |
| Mozambique | 1 |  | 1 |
| Netherlands | 9 |  | 9 |
| Nigeria | 4 |  | 4 |
| Norway | 9 | 1 | 10 |
| Pakistan | 1 |  | 1 |
| Peru | 1 |  | 1 |
| Portugal | 2 |  | 2 |
| Rwanda |  | 1 | 1 |
| South Africa | 4 |  | 4 |
| Spain | 4 |  | 4 |
| Sudan | 4 |  | 4 |
| Sweden | 7 | 3 | 10 |
| Tanzania | 1 |  | 1 |
| Turkey | 6 |  | 6 |
| Uganda | 2 |  | 2 |
| United Kingdom |  | 3 | 3 |
| United States | 28 | 5 | 33 |

**Figure S1:** Map of included study countries


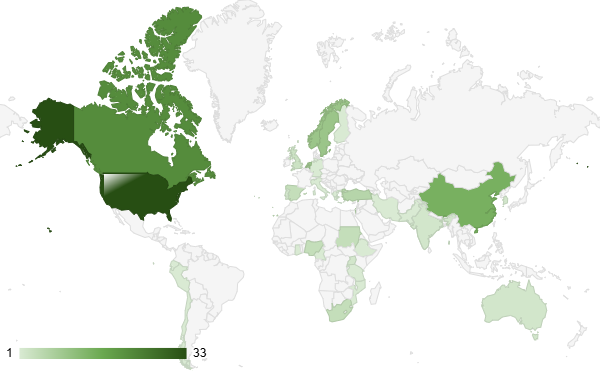

Supplement: figure 1 [file EMS208247-supplement-figure_1.docx]
